# Gender Affirming Hormone Treatment for Trans Adolescents: A Four Principles Analysis

**DOI:** 10.1007/s11673-023-10313-z

**Published:** 2024-01-19

**Authors:** Hane Htut Maung

**Affiliations:** Department of Politics, Philosophy, and Religion, Lancaster University, Lancaster, LA1 4YL United Kingdom

**Keywords:** Gender affirming hormone treatment, Trans adolescents, Four principles of biomedical ethics, Gender dysphoria, Trans healthcare

## Abstract

Gender affirming hormone treatment is an important part of the care of trans adolescents which enables them to develop the secondary sexual characteristics congruent with their identified genders. There is an increasing amount of empirical evidence showing the benefits of gender affirming hormone treatment for psychological health and social well-being in this population. However, in several countries, access to gender affirming hormone treatment for trans adolescents has recently been severely restricted. While much of the opposition to gender affirming hormone treatment for trans adolescents has in part been ideologically motivated, it also reflects a debate about whether there are harms that outweigh the benefits of the treatment. Accordingly, a systematic and comprehensive philosophical analysis of the ethics of gender affirming hormone treatment for trans adolescents is needed. Herein, I offer such an analysis that draws on the four principles of biomedical ethics by Tom Beauchamp and James Childress. Based on the considerations of beneficence, nonmaleficence, autonomy, and justice, I argue that the provision of access to gender affirming hormone treatment for consenting trans adolescents is ethically required and that the current restrictions to such treatment are ethically wrong.

## Introduction

This paper offers a philosophical defence of the provision of gender affirming hormone treatment (GAHT) for trans adolescents. By trans adolescents, I mean young people from the onset of puberty to adulthood who identify as genders other than those to which they were assigned at birth. These include trans girls (young people who identify as girls but were assigned male at birth), trans boys (young people who identify as boys but were assigned female at birth), and nonbinary adolescents (young people who identify neither simply as boys nor as girls).

Gender affirming healthcare encompasses a diverse range of interventions, which can include social affirmation, psychological support and counselling, voice training, gender affirming surgery, and GAHT. In this paper, I focus specifically on GAHT for trans adolescents, which usually involves a combination of puberty blockade (PB) and hormone replacement therapy (HRT). PB involves the use of a gonadotropin releasing hormone analogue to inhibit the endogenous production of oestrogen or testosterone temporarily, which delays the development of secondary sexual characteristics that are incongruent with the young person’s identified gender. HRT involves the exogenous administration of oestrogen or testosterone, which results in the development of secondary sexual characteristics that are congruent with the young person’s identified gender. Because GAHT is usually only prescribed after the onset of puberty, my discussion applies only to pubertal adolescents and not to prepubertal children.

The provision of GAHT for trans adolescents is supported by guidelines from professional organizations such as the World Professional Association of Transgender Health (WPATH) (2022a) and the Endocrine Society (Hembree, et al. 2017). Under these guidelines, the rationale of GAHT is the treatment of gender dysphoria. Accordingly, access to GAHT in most healthcare systems usually requires a diagnosis of gender dysphoria (American Psychiatric Association 2013) or a diagnosis of gender incongruence (World Health Organization 2019). Gender dysphoria refers to the psychological distress that is experienced when one’s identified gender is incongruent with one’s assigned gender at birth. This is a significant problem that can be associated with severe consequences for the health and well-being of trans people, including depression, anxiety, suicidality, and social withdrawal (de Freitas, et al. 2020; Dhejne, et al. 2016; Trevor Project 2020). Furthermore, trans adolescents may be especially vulnerable to these consequences because of how gender dysphoria interacts with physiological puberty and social development (Mason, et al. 2023).

While GAHT is an important part of the care of trans people more generally, I focus specifically on trans adolescents in this paper for two reasons. First, as noted above, the harmful consequences of gender dysphoria in trans adolescents may be further compounded by the changes associated with physiological puberty and social development. Second, gender affirming healthcare has received a large amount of media scrutiny and political hostility in recent years, with much of the antipathy being directed specifically at GAHT for trans adolescents. Subsequently, access to GAHT has recently been restricted in some countries, including the United States, England, and Sweden (Kraschel, et al. 2022; NHS England 2023; Swedish National Board of Health and Welfare 2022). As of June 2023, the National Health Service (NHS) in England has stated that PB for trans adolescents will no longer be routinely available and will only be prescribed as part of clinical research (NHS England 2023).

Much of the opposition to GAHT for trans adolescents has been ideologically motivated. For example, in the United States, the numerous bills that ban certain forms of GAHT reflect a wider trend of political and legal attempts to restrict the rights of LGBTQ+ people. Likewise, in England, the restrictions to GAHT have been situated in a context that includes frequent attacks on trans people by the mainstream media, political opposition to reform of the Gender Recognition Act 2004, and the rise of trans-exclusionary lobby groups, such as Fair Play for Women, LGB Alliance, Sex Matters, and Transgender Trend (Hines 2020; McLean 2021). Accordingly, several professional organizations, including the American College of Physicians (2022), the American Academy of Pediatrics (2022), and WPATH (2022b), have strongly criticized these restrictions to GAHT.

However, some of the opposition to GAHT for trans adolescents has been couched as concerns about the possible harm of such treatment and the quality of the evidence base. For example, the Swedish National Board of Health and Welfare suggests that “the evidence on treatment efficacy and safety is still insufficient and inconclusive” and that “gender confirming treatment thus may lead to a deteriorating of health and quality of life” (Swedish National Board of Health and Welfare 2022, 3–4). Likewise, in its *Interim Service Specification for Specialist Gender Dysphoria Services for Children and Young People* following the closure of the Gender Identity Development Service, NHS England suggests that there is “a lack of evidence to support families in making informed decisions about interventions that may have life-long consequences” (NHS England 2022, 5).

Such criticisms have also been raised in the academic literature. For example, Christopher Richards and colleagues argue that the evidence base for GAHT for trans adolescents is inadequate and suggest that its provision should be curtailed (Richards, et al. 2019). Similarly, Lucy Griffin and colleagues criticize the evidence for GAHT and mention possible harms to bone development, fertility, and future sexual ability (Griffin, et al. 2021). Other critics focus more specifically on the capacities of trans adolescents to make informed decisions. For example, Teresa Baron and Geoffrey Dierckxsens (2022) suggest that meaningful consent cannot be given for potential loss of fertility, while Antony Latham (2022) argues that young people cannot consent to PB because their brains are still developing and their decisions are often coerced by social pressure.

Herein, I argue that these criticisms are unsound. Contrary to the above authors, I suggest that the relevant empirical and moral considerations indicate that the provision of access to GAHT for trans adolescents is ethically required. That is to say, GAHT ought to be available to consenting trans adolescents who seek it as part of their care. Accordingly, I argue that the recent restrictions that have been placed on access to GAHT for trans adolescents are ethically wrong.

The analysis I provide draws on the four principles of biomedical ethics as developed by Tom Beauchamp and James Childress in *The Principles of Biomedical Ethics* (2001). According to this framework, ethical decision-making in healthcare is guided by four principles:

1. Beneficence—the obligation to bring benefit to the person
2. Nonmaleficence—the obligation to avoid harm to the person
3. Autonomy—the obligation to respect the person’s right to self-determination
4. Justice—the obligation to provide just treatment for the person

Each of the four principles can be taken to comprise a prima facie obligation that must be met, although conflicts between the principles can arise in specific cases. When this happens, the respective weights of the obligations must be evaluated (Varkey 2021). Of course, this framework is not without its critics (Cowley 2005; Turner 2003). Nonetheless, it has long been accepted as a prominent philosophical framework for ethical decision-making in healthcare, both with regards to specific cases and to general policies (Gillon 1994).

Previous ethical defences of gender affirming healthcare for trans adolescents have appealed to at least some of the four principles. For example, recent analyses of the benefits and harms of PB (Giordano and Holm 2020) and gender affirming chest surgery (McDougall, et al. 2021) implicitly invoke the considerations of beneficence and nonmaleficence. Perhaps the most extensive ethical analysis of GAHT to date is Florence Ashley’s (2022) defence of adolescent medical transition, which focuses specifically on autonomy but also includes a discussion of why concerns about beneficence and nonmaleficence are sometimes outweighed by this principle. I contribute to this literature by offering a more comprehensive and systematic application of the four principles framework to the ethics of GAHT for trans adolescents. Such an analysis is valuable for the following reasons. First, while it is certainly not exhaustive, the four principles framework brings together diverse consequentialist, deontological, and egalitarian considerations, and so a thorough application of this framework can yield an analysis that covers a sufficiently broad range of issues that are relevant to the debate. Second, as noted above, the four principles framework is widely accepted as the prominent philosophical framework for ethical decision-making in healthcare, and so grounding the analysis in this framework can ensure that it is directly applicable to healthcare practice and policy.

## Beneficence

The principle of beneficence concerns the obligation to act in the benefit of the person:

Promoting the welfare of patients—not merely avoiding harm—expresses medicine’s goal, rationale, and justification … Preventative medicine and active public health interventions have also long embraced concerted social actions of beneficence, such as vaccination programs and health education as obligatory rather than optional. (Beauchamp and Childress 2001, 173)

This is a consequentialist principle, insofar as it suggests that the ethically right course of action is determined by whether it has positive consequences. Here, the relevant question is whether the provision of GAHT has positive consequences for trans adolescents. This can be answered by examining the empirical evidence from clinical research on GAHT.

A large number of studies indicate that GAHT has substantial benefits for the psychological health and social well-being of trans adolescents. The details of these studies are summarized in table 1. Several of these studies found that GAHT in trans adolescents was associated with decreased depression, decreased anxiety, decreased suicidality, and increased body image satisfaction (Allen, et al. 2019; Chen, et al. 2023; Grannis, et al. 2021; Green, et al. 2022; Kuper, et al. 2020; Rew, et al. 2021; Tordoff, et al. 2022; Turban, et al. 2020). Some studies even found that the levels of psychological health and social well-being in trans adolescents who received GAHT improved to levels seen in cis adolescents of similar ages (de Vries, et al. 2014; van der Miesen, et al. 2020). Contrary to the concern raised by Griffin et al. (2021) about the potential effect of GAHT on future sexual ability, a study found that trans people who received GAHT as adolescence reported greater satisfaction with romantic relationships and sexual activities as adults (Bungener, et al. 2020). Indeed, it is possible that such a concern may not be taking into account the variety of sexual activities in which people engage for intimate pleasure, which may not necessarily involve genital contact or penetration (Staples, et al. 2018). Regarding the wider benefits of the provision of GAHT, research by Antoinette Moore (2018) also showed that access to GAHT not only benefited trans adolescents but also had benefits for their families. Therefore, the evidence from these studies indicates that the provision of access to GAHT satisfies the principle of beneficence because it has positive consequences for the health and welfare of trans adolescents.

**Table 1:** Studies on the benefits of puberty blockade (PB) and hormone replacement therapy (HRT) in trans adolescents

**Authors**	**Design**	**Findings**
Allen, et al. 2019	Prospective cohort study of 47 trans adolescents receiving HRT	Trans adolescents who received HRT reported improvements in general well-being and decreased suicidality.
Bungener, et al. 2020	Cross-sectional study of 113 trans people who had received PB and HRT in adolescence and who subsequently received gender affirming surgery in adulthood	Trans people who received PB and HRT in adolescence and gender affirming surgery in adulthood reported improved satisfaction with romantic relationships and sexual activities in adulthood.
Chen, et al. 2023	Prospective cohort study of 315 trans adolescents receiving HRT	Trans adolescents who received HRT reported improvements in appearance congruence, affect, and life satisfaction, and decreases in depression and anxiety. HRT likely decreased depression and anxiety symptoms via the psychological mechanism of improving appearance congruence.
de Vries, et al. 2014	Prospective cohort study of 55 trans people who received PB and HRT in adolescence and who subsequently received gender affirming surgery in adulthood	Trans people who received PB and HRT in adolescence reported improvements in psychological health and social well-being, including decreased depression and anxiety. Trans people who received PB and HRT in adolescence had similar levels of well-being to peers of similar ages from the general population.
Grannis, et al. 2021	Cross-sectional study comparing 19 trans boys who received HRT with 23 trans boys who did not receive HRT	Trans boys who received HRT had significantly lower levels of anxiety, depression, suicidality, and body dissatisfaction than trans boys who did not receive HRT. HRT was correlated with changes in the amygdala-prefrontal circuit that is usually associated with depression and anxiety symptoms.
Green, et al. 2022	Cross-sectional study of 11,914 trans adolescents including those receiving HRT and those not receiving HRT	Trans adolescents who were receiving HRT had significantly lower rates of depression and suicidality than trans adolescents who were not receiving HRT.
Kuper, et al. 2020	Prospective cohort study of 148 trans adolescents receiving PB and HRT	Trans adolescents who received PB and HRT reported improvements in depression, anxiety, and body dissatisfaction.
Moore 2018	Prospective cohort study of 179 trans adolescents receiving gender affirming healthcare including PB and HRT	Trans adolescents who received gender affirming healthcare exhibited significant improvements across multiple domains of well-being, with the greatest improvements seen after PB and HRT were commenced. The families of the trans adolescents also reported improved well-being.
Rew, et al. 2021	Research review of 9 studies on PB in trans adolescents	PB for trans people in adolescence is associated with improved psychological health, improved social well-being, and decreased suicidality in adulthood.
Tordoff, et al. 2022	Prospective cohort study of 104 trans adolescents including 69 receiving PB or HRT and 35 not receiving PB or HRT	Trans adolescents who received PB or HRT had significantly lower rates of depression and suicidality than trans adolescents who did not receive PB or HRT
Turban, et al. 2020	Cross-sectional study of 20,619 trans adolescents including those receiving PB and those not receiving PB	Trans adolescents who were receiving PB had lower odds of suicidality than trans adolescents who were not receiving PB
van der Miesen, et al. 2020	Cross-sectional study comparing 178 trans adolescents receiving PB, 272 trans adolescents not receiving PB, and 651 cis adolescents	Trans adolescents who were receiving PB had better psychological well-being than trans adolescents who were not receiving PB and similar psychological well-being to cis adolescents of similar ages.

A common objection is that the above evidence is of low quality because the studies are methodologically suboptimal (NHS England 2022; Swedish National Board of Health and Welfare 2022). For example, Richards et al. suggest that the provision of PB “should be curtailed until we are able to apply the same scientific rigour that is demanded of other medical interventions” (Richards, et al. 2019, 611). Similarly, Griffin et al. (2021) note that research on GAHT in trans adolescents does not make randomized controlled comparisons. Rather, much of the evidence on GAHT in trans adolescents comes from cross-sectional studies and longitudinal cohort studies. The randomized controlled trial (RCT) is often considered to comprise the gold standard for evidence-based medicine because the randomization of participants into intervention and control groups facilitates a fair comparison of the outcomes of intervening and not intervening. In their reviews of the evidence regarding GAHT, the National Institute for Health and Care Excellence (NICE) applied the Grading of Recommendations Assessment, Development, and Evaluation framework for evaluating evidence (Guyatt, et al. 2008). Under this framework, RCT evidence is prima facie considered to be of higher certainty than evidence from observational studies. Accordingly, NICE (2022a; 2022b) judged the evidence for the benefits of GAHT to be of “low certainty.”

In response, I argue that the available evidence is still sufficient to support the benefits of GAHT for trans adolescents in spite of the lack of RCT data. First, it is worth noting that the reviews by NICE (2022a; 2022b) do not include several important studies which demonstrate positive outcomes. As shown in table 1, there are now more methodologically robust studies on GAHT that use large sample sizes (Green, et al. 2022), use appropriate comparison groups (Tordoff, et al. 2022; van der Miesen, et al. 2020), and yield mechanistic evidence (Chen, et al. 2023; Grannis, et al. 2021), which were not considered in the reviews. Therefore, the reviews by NICE do not represent the most up-to-date evidence on the benefits of GAHT.

Second, as recently argued by Ashley et al. (2023), evidence from RCTs is often not required of many other medical interventions. Indeed, the valorization of RCTs in evidence-based medicine has been extensively criticized for neglecting other important sources of evidence (Anjum and Mumford 2017; Deaton and Cartwright 2018; Frieden 2017; Grossman and Mackenzie 2005). For some interventions, RCTs are ethically unfeasible because the consequences of not intervening would be unacceptably harmful. A famous example is the lack of RCT evidence for the effectiveness of parachute use to prevent death (Yeh, et al. 2018). Another example, discussed by Ashley et al. (2023), is the lack of RCT evidence for the psychological health benefits of providing access to abortion. Here, an RCT would be ethically unfeasible due to the harmfulness of making participants in the control group proceed with unwanted pregnancies. Nonetheless, it is widely accepted that the provision of access to abortion is clinically and ethically justified. An analogous point could be made regarding GAHT for trans adolescents, where an RCT would harm participants in the control group by making them develop secondary sexual characteristics that are incongruent with their identified genders. Such an RCT would also be methodologically unfeasible because the physiological effects of GAHT would be so apparent that blinding could not be maintained. In such situations where RCT evidence is not available, clinical decisions are commonly informed by other forms of evidence, as exemplified by the case-control study evidence that a supine sleeping position reduces the risk of sudden infant death syndrome (Mitchell et al. 1991) and the epidemiological evidence that mask use mitigates COVID-19 transmission (Pearce and Vanderbroucke 2021). Therefore, contrary to what critics claim, GAHT for trans adolescents is based on “the same scientific rigour that is demanded of other medical interventions” (Richards, et al. 2019, 611).

Third, even though the evidence on GAHT for trans adolescents does not come from RCTs, it is nonetheless sufficient to support the causal hypothesis that GAHT has benefits for the psychological health and social well-being of trans adolescents. As Federica Russo and Jon Williamson (2007) note, the assessment of causation in healthcare requires two kinds of evidence: (1) probabilistic evidence, and (2) mechanistic evidence. Probabilistic evidence is usually gathered by establishing a statistical correlation between an intervention and a given outcome that holds across different studies, such that this outcome is consistently more likely to obtain in the presence of the intervention than in its absence. This can be understood through James Woodward’s (2003) interventionist theory of causation, which states that *C* is a cause of *E* if there is a possible intervention on *C* that makes a difference to *E*. Mechanistic evidence consists of an empirically informed theoretical explanation of how intervening on *C* makes a difference to *E*.

The results from the aforementioned studies on GAHT for trans adolescents are sufficient to establish these two kinds of evidence. With respect to probabilistic evidence, some of the studies summarized in table 1 had control groups that enabled comparisons of the outcomes for trans adolescents who received GAHT and those who did not (Green, et al. 2022; Tordoff, et al. 2022; Turban, et al. 2020). Of these studies, one was a prospective cohort study that allowed an assessment of the temporal relation between the intervention and the outcome (Tordoff, et al. 2022) while another was a cross-sectional study that minimized the margin of error by studying a large sample of 11,914 participants (Green, et al. 2022). With respect to mechanistic evidence, some of the studies summarized in table 1 highlighted some social, psychological, and biological mechanisms through which GAHT decreases depression and anxiety (Chen, et al. 2023; Grannis, et al. 2021). And so, based on the above, there is sufficient probabilistic and mechanistic evidence to support the claim that GAHT has positive consequences for trans adolescents. This evidence indicates that the provision of access to GAHT for trans adolescence satisfies the principle of beneficence.

## Nonmaleficence

The principle of nonmaleficence concerns the obligation to minimize harm to the person:

In medical ethics it has been closely associated with the maxim *Primum non nocere*: “Above all [or first] do no harm” … the Hippocratic oath clearly expresses an obligation of nonmaleficence and an obligation of beneficence: “I will use treatment to help the sick according to my ability and judgment, but I will never use it to injure or wrong them.” (Beauchamp and Childress 2001, 113)

Again, this is a consequentialist principle, insofar as it suggests that the ethically right course of action is determined by whether it avoids negative consequences. However, when assessing nonmaleficence, a reasonable threshold must be assumed. Almost all medical interventions are associated with risks of adverse outcomes, many of which we consider to be acceptable to tolerate (Ashley 2022). Hence, the relevant question here is whether GAHT for trans adolescents has negative consequences whose likelihood and severity are so great that they make the intervention ethically impermissible.

In general, the evidence to date indicates that GAHT for trans adolescents is reasonably safe. As noted above, research shows that GAHT does not cause overall psychological harm but rather is associated with improved psychological health and social well-being in trans adolescents (Allen, et al. 2019; Bungener, et al. 2020; Chen, et al. 2023; de Vries, et al. 2014; Grannis, et al. 2021; Green, et al. 2022; Kuper, et al. 2020; Moore 2018; Rew, et al. 2021; Tordoff, et al. 2022; van der Miesen, et al. 2020). Regarding physiological health, research has indicated that the safety profile of GAHT is favourable when compared with other medical interventions that we usually deem to be acceptable (Chew, et al. 2018; Mahfouda, et al. 2017). There are some increased risks of venous thromboembolism with oestrogen therapy and of erythrocytosis with testosterone therapy. However, by performing regular monitoring, modifying the route of administration, and addressing other comorbidities, these risks can be mitigated so that they are comparable to the risks for cis women who are taking combined oral contraceptives and for cis men with hypogonadism who are taking testosterone (Kotamarti, et al. 2021; Madsen, et al. 2021). Supporting this, a recent epidemiological study in England found that the increased mortality among trans people compared with cis people is due to reasons that are unrelated to GAHT (Jackson, et al. 2023).

Nonetheless, despite the evidence of its general safety, critics have raised concerns about some specific risks of GAHT for trans adolescents. The first concern, briefly raised by Griffin et al. (2021), is the risk of osteoporosis with PB. Studies have suggested that trans adolescents who receive PB have decreased bone density compared to cis adolescents of similar ages (Carmichael, et al. 2021; Schagen, et al. 2020). This is due to the lack of oestrogen and testosterone, which usually have roles in bone development. However, it is uncertain whether the decreased bone density is associated with any clinically significant problems for subsequent bone health. Furthermore, bone density has been shown to increase when HRT is commenced (Klink, et al. 2015; Vlot, et al. 2017). Therefore, the risk of osteoporosis can be mitigated by avoiding the delay in commencing HRT for trans adolescents who wish to proceed with medical transition, as well as by addressing other potential contributory factors such as diet, sunlight exposure, and activity level.

The second concern, raised by Baron and Dierckxsens (2022) and briefly by Griffin et al. (2021), is the risk of infertility. Fertility is often maintained in trans people who commence GAHT in adulthood, but the impact of treatment on the fertility of trans people who commence GAHT in adolescence is less clear. With PB, the effect is reversible, and so fertility can be expected to be restored if the treatment is stopped. With HRT, the impact on future fertility may depend on the stage of puberty at which treatment is commenced, as trans adolescents who commence GAHT in early in puberty may not yet have mature gametes (Cheng, et al. 2019).

However, while the possible impact of GAHT on fertility is certainly an important risk that warrants serious consideration, I argue that the presence of this risk does not justify banning GAHT for trans adolescents. There are other medical interventions in childhood that negatively impact future fertility but are deemed to be acceptable, such as chemotherapy and radiotherapy to treat paediatric cancers. In these cases, the interventions are lifesaving and so the harms of providing treatment are outweighed by the harms of not providing treatment. Likewise, I argue that untreated gender dysphoria can have a similar sort of urgency. Gender dysphoria in trans adolescents has been shown to be associated with severe consequences, including attempted suicide, self-harm, and deteriorating psychological health (Tan, et al. 2023; Trevor Project 2020). Moreover, as discussed earlier, there is evidence that access to GAHT in trans adolescents can mitigate these consequences (Allen, et al. 2019; Bungener, et al. 2020; Chen, et al. 2023; de Vries, et al. 2014; Grannis, et al. 2021; Green, et al. 2022; Kuper, et al. 2020; Moore 2018; Rew, et al. 2021; Tordoff, et al. 2022; van der Miesen, et al. 2020). And so, given the severe consequences of untreated gender dysphoria, GAHT in trans adolescents can be considered a lifesaving intervention whose risk to fertility is outweighed by the harms of not providing the treatment.

Furthermore, it should be noted that WPATH’s (2022b) international standards of care have specific guidelines for addressing the risk to fertility when offering GAHT for trans adolescents:

We recommend health care professionals working with transgender and gender diverse adolescents requesting gender-affirming medical or surgical treatments inform them, prior to the initiation of treatment, of the reproductive effects, including the potential loss of fertility and available options to preserve fertility within the context of the youth's stage of pubertal development … Parents are advised to be involved in this process and should also understand the pros and cons of the different options … HCPs should specifically pay attention to the developmental and psychological aspects of fertility preservation and decision-making competency for the individual adolescent. (WPATH 2022a, S57)

Hence, the potential risk to fertility is taken seriously. The guidelines explicitly recommend that trans adolescents who are considering GAHT must be informed about the possible impact on fertility so that they can evaluate whether this is outweighed by the benefits of proceeding with GAHT and the harms of not proceeding with GAHT. Moreover, it is recommended that trans adolescents and their families are counselled about the options for fertility preservation and about the different possible paths to parenthood before commencing GAHT, which could partially mitigate some of the concerns about future fertility. Indeed, research suggests that trans people who intend to become parents wish to do so through a variety of paths, including birth, surrogacy, adoption, and fostering (Brown 2021).

The third concern, raised by Baron and Dierckxsens (2022) and Latham (2022), is the risk of regret. Here, the worry is that the gender identities of adolescents may not yet be fully formed, and so some adolescents who choose to commence GAHT may later regret their choices. This is a concern that has recently received significant attention in the mainstream media (Singal 2018). A notable case in England was that of Keira Bell (2021), who received GAHT at the Tavistock and Portman NHS Foundation Trust as an adolescent and subsequently regretted the decision. A legal complaint was lodged and in 2020 the High Court ruled against the Tavistock and Portman NHS Foundation Trust. However, in 2021 the Court of Appeal overturned this ruling and suggested that adolescents aged under sixteen can consent to PB.

This case highlights that some adolescents do indeed regret receiving GAHT. However, the risk of harm from regret is not unique to GAHT but is a risk that is associated with all medical interventions. Research suggests that a substantial proportion of people who receive common interventions, such as knee replacement surgery and radical prostatectomy, subsequently express regret, yet providing access to these interventions is considered acceptable (Kahlenberg, et al. 2018; Ratcliff, et al. 2013). Accordingly, I argue that the risk of harm from regret does not justify restricting access to GAHT, as this would result in harms to many more trans adolescents who are suffering from untreated gender dysphoria. Rather, the presence of the risk of regret warrants procedures being implemented to ensure that people seeking GAHT appreciate this risk, as well as to ensure that people who do regret GAHT are provided with care and support (MacKinnon, et al. 2022).

It is also important to put the magnitude of risk into context, as the regret rate for GAHT in trans adolescents is lower than commonly assumed. Early research by Kenneth Zucker and Susan Bradley (1995) suggested that the majority of children who present with gender nonconformity later revert to their genders assigned at birth when they reach adolescence. Similar results were also reported by subsequent studies (Drummond, et al. 2008; Wallien and Cohen-Kettenis 2008). However, methodological problems with these studies have been raised which suggest that they cannot reliably support inferences about the regret rate for GAHT. Notably, the studies examined the persistence of gender noncomformity in prepubertal children, which is a poor proxy for the stability of gender identity or the desire for medical transition in pubertal adolescents (Ashley 2021). It has also been noted that the diagnostic criteria used by these studies were overinclusive and did not distinguish trans identity from other kinds of gender nonconformity (Temple Newhook, et al. 2018). Accordingly, there are substantial disparities between the results of these studies and the results of research which focus more specifically on gender identity. For example, a recent study of 317 trans adolescents found that five years after socially transitioning, 94 per cent had maintained their male or female trans identities, 3.5 per cent had proceeded to identify as nonbinary, and only 2.5 per cent had reverted to their previous cis identities (Olson, et al. 2022). This suggests that gender identity in pubertal adolescents is reasonably stable.

Clinical research also indicates that the regret rate for GAHT in trans adolescents is very low. An early study of twenty-two trans people who received GAHT in adolescence found that none of the participants in adulthood regretted the decisions they had taken (Cohen-Kettenis and van Goozen 1997). More recently, two studies respectively found that 96.5 per cent and 100 per cent of trans adolescents who initiated PB were subsequently happy to proceed with HRT at a later stage (Brik, et al. 2020; de Vries, et al. 2011). Another recent study of 720 trans people who commenced HRT in adolescence also found that 98 per cent of participants chose to continue HRT into adulthood (van der Loos, et al. 2022). Importantly, research on trans adults who chose to detransition has shown that the predominant reasons why the people decide to detransition are external problems such as family rejection and social stigma, rather than regret (Turban, et al. 2021).

Of course, none of the above denies that adolescents who regret receiving GAHT are harmed by it. However, it does indicate that the overall regret rate for GAHT for trans adolescents is much lower than commonly assumed. Indeed, as noted above, the risk of regret for GAHT appears to be substantially lower than for other healthcare interventions which are usually considered to be acceptable (Kahlenberg, et al. 2018; Ratcliff, et al. 2013). And so, when weighed against the benefits of treatment and the harms of withholding treatment, the low risk of regret is insufficient to justify restricting access to GAHT for trans adolescents. Nonetheless, it is recommended that trans adolescents are informed about the possible risk of future regret and are supported if they do exhibit regret.

The above also raises a more general point about nonmaleficence. When assessing the potential harm of providing an intervention, this must be evaluated relative to the potential harm of withholding the intervention. The decision to withhold GAHT is not a neutral decision but is a decision that has major and enduring consequences for trans adolescents. As noted by WPATH, “allowing irreversible puberty to progress in adolescents who experience gender incongruence is not a neutral act given that it may have immediate and lifelong harmful effects for the transgender young person” (WPATH 2022a, S48). Research has shown that lack of access to GAHT for trans adolescents is associated with higher rates of mental illness and attempted suicide (Tan, et al. 2023; Tordoff, et al. 2022). Furthermore, in a recent study, trans adolescents who had GAHT delayed until they were older were shown to suffer from significantly higher rates of depression and suicidality than trans adolescents who received GAHT promptly (Sorbara, et al. 2020).

And so, the potential negative consequences associated with GAHT are greatly outweighed by the negative consequences of withholding GAHT. This indicates that the decision to provide GAHT for trans adolescents satisfies the principle of nonmaleficence when evaluated relative to the harms of restricting access to treatment. Furthermore, given how considerable these harms are, I argue that the current restrictions to access to GAHT for trans adolescents are ethically wrong because they fail to satisfy the principle of nonmaleficence.

## Autonomy

The principle of autonomy concerns the obligation to respect the person’s right to self-determination:

To respect an autonomous agent is, at a minimum, to acknowledge that person’s right to hold views, to make choices, and to take actions based on personal values and beliefs … Respect, on this account, involves acknowledging decision-making rights and enabling persons to act autonomously, whereas disrespect for autonomy involves attitudes and actions that ignore, insult, or demean others’ rights of autonomy. (Beauchamp and Childress 2001: 63)

This is a deontological principle, insofar as it acknowledges that one has unconditional worth as an agent who has the right to govern oneself, make choices, and act according to one’s values. As the above passage shows, the issue that Beauchamp and Childress take to be relevant here is “respect for autonomy,” which involves acknowledging and enabling the right to exercise self-determination. Hence, the relevant question here is whether the provision of GAHT for trans adolescents respects their rights to govern themselves as autonomous agents.

Autonomy has been characterized in various ways in the philosophical literature, but Beauchamp and Childress defend a nonideal account:

We analyze autonomous action in terms of normal choosers who act (1) intentionally, (2) with understanding, and (3) without controlling influences that determine their action … acts can satisfy both the conditions of understanding and absence of controlling influences to a greater or lesser extent. (Beauchamp and Childress 2001, 59)

Such an account recognizes that acts may be autonomous by degrees. This is especially relevant to the discussion of GAHT for trans adolescents, as young people may vary with regards to their understandings and external controlling influences. Hence, it acknowledges that different people may be differently situated to make autonomous decisions.

As noted above, self-determination is usually considered to be a key aspect of autonomy. In the clinical context, this is often connected with the notions of decisional capacity and informed consent. Hence, Beauchamp and Childress focus their discussion of autonomy on “individuals’ decision-making in health care and research, especially informed consent and refusal” (Beauchamp and Childress 2001, 57). Decisional capacity is commonly understood as “the degree to which an individual has the ability to understand a proposed therapy or procedure, including its risks, benefits, and alternatives; to communicate relevant questions; and to arrive at a decision consistent with his or her values” (Cummings and Mercurio 2010, 252). Moreover, the level of capacity required to consent to an intervention is usually considered to be proportional to the potential seriousness of the intervention.

A detailed defence of GAHT on the basis of autonomy is provided by Ashley (2022), who draws an analogy with reproductive healthcare. As Ashley notes, “birth control, abortion, and adolescent medical transition are analogous insofar as they intervene on healthy physiological states such as puberty, sexual traits, fertility, and pregnancy, by reason of the person’s fundamental self-conception and desired life” (Ashley 2022, 128). Given that gender is very often an important aspect of one’s personal identity and how one relates to the world, philosophers such as Talia Mae Bettcher (2009) and Lori Watson (2016) have argued that there is an obligation to respect one’s first-person authority over one’s gender identity. Likewise, the lawyer Jillian Weiss (2001) has proposed that one’s gender identity falls within the sphere of one’s autonomy and right to privacy. Accordingly, the provision of GAHT for trans adolescents respects their rights as autonomous agents, insofar as it enables them to affirm their identities and fulfil their embodiment goals. Furthermore, restricting access to GAHT amounts to a violation of one’s autonomy because it would cause one to undergo a puberty that is incongruous with one’s gender identity.

However, opponents of GAHT have objected that trans adolescents may not have the relevant understandings to make autonomous decisions. For example, Baron and Dierckxsens (2022) suggest that young people cannot meaningfully consent to GAHT because they may not fully understand the implications of the potential loss of fertility. They note that “however objectively such risks and probabilities are presented, this process is value-laden from the perspective of all involved” (Baron and Dierckxsens 2022, 605). Similarly, Latham (2022) suggests that young people do not have the cognitive capacities to consent to GAHT because their brains are still developing. This objection was also key part of the aforementioned legal action against the Tavistock and Portman NHS Foundation Trust.

In response, I argue that the above objection is unsound. First, as I shall discuss later, there is empirical evidence which suggests that many trans adolescents do exhibit the appropriate understandings and capacities to make decisions about GAHT (Clark and Virani 2021). Second, the objection that the process of obtaining consent from young people is “value-laden” assumes an inappropriately stringent conception of consent that is unfeasible for assessing autonomy in the clinical context. As noted above, Beauchamp and Childress reject an ideal conception of autonomy and instead endorse a nonideal account:

For an action to be autonomous in this account, it needs only a substantial degree of understanding and freedom from constraint, not a full understanding or a complete absence of influence. To restrict adequate decision-making by patients and research subjects to the ideal of fully or completely autonomous decision-making strips their acts of any meaningful place in the practical world, where people’s actions are rarely, if ever, fully autonomous. (Beauchamp and Childress 2001, 59)

This nonideal account recognizes that the decisions made by competent agents are ordinarily influenced by various epistemic constraints and values, and so the appropriate standard for what comprises a required level of understanding for an autonomous act must accommodate these epistemic constraints and values. Indeed, people often make major decisions which are considered autonomous even though their values may later change in unforeseen ways and their understandings of the implications of their decisions are limited. For example, L. A. Paul (2014) notes that one’s decision to have a child cannot be based on full understanding because becoming a parent is such a transformative experience that one is not epistemically situated to understand how it will impact one’s future values and desires. This also applies to many routine medical interventions that alter people’s physiological and psychological capacities, including orthopaedic surgery, weight loss treatment, and psychiatric medication.

Given that adults are not ordinarily expected to have full understandings of how such decisions will impact their future values and desires, it would be unreasonable to expect trans adolescents to have full understandings of how the potential loss of fertility will impact their future values and desires. Under the nonideal account of autonomy endorsed by Beauchamp and Childress (2001), a trans adolescent who has reasonable knowledge and intellectual appreciation of the risk may still have the required level of understanding for the decision GAHT to be autonomous. Indeed, given trans adolescents’ personal experiences of gender dysphoria and of critically examining their own identities, there are respects in which they are better situated to understand the implications of GAHT than cis people who have not struggled with these challenges. As Timothy Murphy notes, “one may be hard pressed to make the case that reflective and informed 16-year-olds or 17-year-olds who have experienced gender dysphoria most of their lives necessarily lack the cognitive wherewithal to commit themselves to body modifications having durable effects” (Murphy 2019, 638).

Of course, as noted above, young people may vary with regards to their understandings and external controlling influences. However, this does not warrant blanket restrictions on GAHT for trans adolescents. Rather, it warrants procedures and safeguards to ensure that decisional capacity is demonstrated and that informed consent is obtained. Accordingly, the clinical guidelines by WPATH (2022a) and the Endocrine Society (Hembree et al. 2017) explicitly recommend that a young person who is seeking GAHT must demonstrate the emotional and cognitive maturity required to provide informed consent for the treatment. Furthermore, according to Beauchamp and Childress, a key aspect of respect for autonomy is “enabling persons to act autonomously” (Beauchamp and Childress 2001, 63). This indicates that young people ought to be supported to gain the understandings required to make autonomous decisions about their healthcare, such as through counselling, education, and family support. Hence, as Murphy proposes, trans adolescents’ “characterizations of their gender should be taken as the starting point for any healthcare offered to them and their choices respected in a way scaled to their decision-making capacities” (Murphy 2019, 639).

A related objection, raised by Latham (2022), is that trans adolescents’ decisions may not be autonomous because they are influenced by social pressure from their peers. This objection draws on research by Lisa Littman (2018), which recruited parents of young people with gender dysphoria through three websites and asked them to complete questionnaires. The reports from the parents suggested that substantial proportions of the young people had friendship groups where most people were trans, showed increases in social media usage after expressing gender dysphoria, exhibited worsening relationships with their families after expressing gender dysphoria, and suffered from various mental health conditions. The hypothesis was that these young people had a form of “rapid-onset gender dysphoria” that was caused by social pressure.

However, the methodology of Littman’s (2018) study has been widely criticized and, shortly after the study was published, *PLoS One* conducted a post-publication review which later led to the publication of a correction to the paper (Littman 2019). Notably, the study only sought the testimonies of parents but did not seek the testimonies of the young people themselves, who may have been epistemically better situated to describe their gender identities and may have provided very different accounts of their circumstances (Restar 2020). Moreover, the study recruited parents through trans-exclusionary websites, such as Transgender Trend and Youth Trans Critical Professionals, which raises serious concerns about sampling bias and testimonial credibility (Ashley 2020). The study has also been criticized by Ashley (2020) for drawing the wrong conclusions from the findings about mental health conditions, relationships with families, and friendship groups. For example, worsening mental health does not necessarily indicate that one is unhappy with one’s trans identity but rather is often the outcome of family rejection for being trans (Klein and Golub 2016). Also, the finding that the young people had friendship groups where most people are trans does not necessarily indicate social pressure but could just indicate that young people seek friendship groups and social media platforms which allow them to explore and express their gender identities safely.

The social pressure hypothesis has also been undermined by subsequent research. For example, recent research by Beth Clark and Alice Virani (2021) has shown that trans adolescents aged between fourteen and eighteen readily demonstrated the relevant understandings and abilities that mark the capacity to consent to GAHT. As well as being able to understand the interventions and alternatives, evaluate the risks and benefits, and make decisions consistent with their values, the research showed that trans adolescents were able to reflect critically on their gender identities and their own responsibilities regarding their healthcare decisions. Therefore, contrary to Latham’s (2022) claim about the influence of social pressure, the evidence indicates that the decisions of many trans adolescents to commence GAHT are not impulsive or coerced by social pressure from their peers, but generally result from thoughtful and extensive deliberation about their identities, values, and desires.

Of course, this is not to deny that such decisions are influenced by social factors. We reside in a society where gender is a central organizing classification system, and so it is inevitable that social factors influence how we construct our gender identities. However, the presence of social influence does not preclude authenticity. As beings who are socially embedded, many of our desires are profoundly influenced by our social interactions but are nonetheless endorsed by us authentically. For example, a person’s desire to have a child may be partly influenced by the pronatalist society wherein the person resides but may still be endorsed authentically and autonomously by the person (Maung 2019). Hence, what is relevant here with respect to autonomy is not the source of the desire but whether the decision is endorsed by the person and not coerced.

Another objection that has been raised against GAHT is that allowing young people to consent to GAHT would be analogous to abolishing the legal age of sexual consent. For example, a recent opinion piece in *The Telegraph* suggested that the law does not allow children to consent to sexual activity, and so it should not allow them to consent to GAHT (Williams 2020). However, in response, I argue that this objection misconstrues the purpose of the legal age of sexual consent. The purpose of the legal age of sexual consent is not to suggest that adolescents cannot make autonomous decisions but is to protect children from sexual abuse and exploitation, mostly by adults (Fleming 2006). Some legislation, such as the Sexual Offences Act 2003 in England and Wales, sets a minimum legal age of consent but also recognizes that mutually consensual sexual activity between adolescents does take place and that “the law is not intended to prosecute mutually agreed teenage sexual activity between two young people of a similar age, unless it involves abuse or exploitation” (Home Office 2004, 3). This is also reflected in the close-in-age exemption laws in some other jurisdictions, which aim to prevent adolescents who are in mutually consensual sexual relationships from being prosecuted but which still recognize that younger children are unable to give sexual consent and that there is a need to protect children from abuse by adults (Fix-Cañas, et al. 2022; Kanbur 2019). And so, while it is recognized that sexual contact between an adult and a child is always wrong because of the serious harm associated with exploitation and violation, there is also some recognition that mutual and nonexploitative sexual activity between close-in-age adolescents who are near or around the legal age of sexual consent can be consensual and morally permissible. The fact that the legal age of sexual consent prohibits the sexual abuse and exploitation of children does not imply that adolescents are incapable of making autonomous decisions.

Indeed, adolescents are often trusted to make autonomous decisions about other aspects of their healthcare beyond GAHT. As Murphy notes, “maturing adolescents may make healthcare decisions for themselves in regard to prenatal care, terminating a pregnancy, entering clinical research trials, organ donation, and even life-saving treatment” (Murphy 2019, 637–638). Furthermore, there are legal frameworks which protect the rights of adolescents to consent to such interventions without parental permission and the duties of clinicians to maintain confidentiality unless exploitation is suspected, such as Gillick competence and Fraser guidelines in England and Wales (Fleming 2006; Free 2005; Larcher and Hutchinson 2010). There is no reason why trans adolescents’ rights to consent to GAHT should not also be protected under frameworks such as these.

## Justice

The principle of justice concerns the obligation to provide just care for the person:

Many barriers exist to achieving access to health care. For millions who encounter these barriers, a just health care system remains a distant goal. Even though every society must ration access to health care through some mechanism(s), many can close gaps in access more conscientiously than they have to date … we have proposed that society recognize an enforceable right to a decent minimum of health care within a framework for allocation that incorporates both utilitarian and egalitarian standards. (Beauchamp and Childress 2001: 272).

As noted above, this is partly an egalitarian principle, insofar as it emphasizes that members of society should be treated equitably. The relevant question here is whether the provision of GAHT for trans adolescents is a just use of healthcare.

Of course, justice is a broad and multifaceted notion which has been interpreted in various ways in the philosophical literature. Accordingly, Beauchamp and Childress suggest that “no single theory of justice or system of distributing health care is necessary or sufficient for constructive reflection on policy” (Beauchamp and Childress 2001, 272). Nonetheless, a particular aspect of justice which is discussed by Beauchamp and Childress is the obligation to address the health disparities that impact marginalized groups. For example, they note that healthcare in the United States has been distributed unfairly based on gender and race, “resulting in a differential impact on women and minorities” (Beauchamp and Childress 2001, 238). This aspect of justice is also especially relevant to the discussion of GAHT for trans adolescents, who are also differentially impacted by healthcare injustices.

Research indicates that trans people suffer significant health disparities compared to cis people, including poorer psychological health, poorer social well-being, poorer care utilization, increased suicidality, and increased mortality (Jackson, et al. 2023; Rider, et al. 2018; Safer, et al. 2016). These health problems are not intrinsic to being trans, but are occasioned by various injustices, including family rejection, transphobic violence, social stigma, vilification by the media, and exclusionary social policies (Katz-Wise, et al. 2018; Klein and Golub 2016; Hughto, et al. 2015; Zwickl, et al. 2021). In addition to these, lack of access to gender affirming healthcare has been shown to be a significant factor that contributes to the aforementioned health disparities. For example, recent research has indicated that trans adolescents without access to GAHT have significantly higher rates of depression and suicidality than trans adolescents with access to GAHT and their cis peers (Tan, et al. 2023; Tordoff, et al. 2022).

Given the above, the restriction of access to GAHT comprises an injustice, insofar as it exacerbates health disparities between trans people and cis people. Indeed, there are several indicators that access to GAHT for trans adolescents is an unmet healthcare need. In the United Kingdom, the average waiting time for a trans adolescent to receive treatment at an NHS gender clinic is three years, which is well in excess of the agreed target of eighteen weeks (Good Law Project 2023). Moreover, it has been reported that doctors are often reluctant to prescribe GAHT for trans patients (Barratt 2016). In a recent study in Aotearoa, GAHT was reported as an unmet need by 42 per cent of trans adolescents (Tan, et al. 2023). Due to the inaccessibility of gender affirming healthcare, many trans people have turned to “do-it-yourself” HRT by obtaining hormones from unregulated sources (Baker, et al. 2023). Also, as noted by Hil Malatino (2020), trans people have had to develop their own informal care networks due to the challenges of accessing treatment in formal care settings.

These barriers reflect a double standard for access to GAHT and access to other sorts of healthcare. As noted by Miranda Fricker and Katharine Jenkins (2017), trans people often suffer testimonial injustices in the healthcare setting. That is to say, they are often granted less authority over their experiences of gender incongruence than people usually are over other sorts of experiences. As Ashley (2019) notes, when someone says “my arm hurts,” clinicians typically trust that claim and would be acting unjustly if they do not, yet trans people are often made to feel that they have to “prove” to clinicians that they are “really” trans. To access GAHT, trans people usually have to undergo lengthy and burdensome psychological assessments that can last several months (Schulz 2018). Such lengthy psychological assessments are generally not required for access to other hormonal interventions, such as hormonal contraception, hormonal control of menstruation, testosterone for hypogonadism in cis men, and oestrogen and progesterone for the menopause in cis women. This is despite the fact that these interventions involve the same hormones as GAHT.

And so, the provision of access to GAHT for trans adolescents is a just use of healthcare resources for two reasons. First, given that lack of access to GAHT is a contributor to health disparities between trans adolescents and their cis peers, providing access to GAHT for trans adolescents can help to address these disparities so that healthcare resources are distributed fairly according to clinical need. Second, improving the provision of access to GAHT would help to correct the unfair double standard that is currently enforced for access to GAHT and access to other sorts of healthcare. This would satisfy the considerations noted by Beauchamp and Childress of closing “gaps in access more conscientiously” and helping to ensure “a decent minimum of health care” (Beauchamp and Childress 2001, 272).

## Conclusion

Herein, I have applied the philosophical framework of the four principles of biomedical ethics (Beauchamp and Childress 2001) to argue that the provision of access to GAHT for trans adolescents is ethically required. Regarding beneficence, the available evidence to date indicates that the provision of access to GAHT is associated with substantial benefits for the psychological health and social well-being of trans adolescents. Regarding nonmaleficence, research indicates that the potential risks of providing access to GAHT are greatly outweighed by the considerable harms of restricting access to GAHT. Regarding autonomy, the provision of access to GAHT respects the rights of trans adolescents to govern themselves as autonomous agents. Regarding justice, the provision of access to GAHT is a just use of healthcare that can help to alleviate the considerable health disparities suffered by trans adolescents.

The analysis I have provided suggests that the opposition to GAHT for trans adolescents is unsound. Contrary to the concerns raised by academic critics (Baron and Dierckzsens 2022; Griffin, et al. 2021; Latham 2022; Richards, et al. 2019), journalists (Singal 2018; Williams 2020), and governmental agencies (NHS England 2022; Swedish National Board of Health and Welfare 2022), there is sufficient evidence that access to GAHT has significant benefits which outweigh the potential harms and that trans adolescents are often situated to make informed decisions about their healthcare. I have argued that the current restrictions to GAHT for trans adolescents are ethically wrong because they result in considerable psychological and social harms, contribute to health injustices, and undermine the rights of young people to determine their own identities. Therefore, I conclude that GAHT ought to be available to consenting trans adolescents who wish to receive it as part of their care.

## Data Availability

Not applicable

## References

[CR1] Allen, L.R., L.B. Watson, A.M. Egan, and C.N. Moser. 2019. Well-being and suicidality among transgender youth after gender-affirming hormones. *Clinical Practice in Pediatric Psychology* 7(3): 302–311.

[CR2] American Academy of Pediatrics. 2022. Statement from the American Academy of Pediatrics and the Oklahoma Chapter of the American Academy of Pediatrics on gender-affirming care. https://www.aap.org/en/news-room/news-releases/aap/2022/statement-from-the-american-academy-of-pediatrics-and-the-oklahoma-chapter-of-the-american-academy-of-pediatrics-on-gender-affirming-care/#:~:text=The%20American%20Academy%20of%20Pediatrics%2C%20along%20with%20its%20Oklahoma%20Chapter,transgender%20and%20gender%20diverse%20youth. Accessed May 9, 2023.

[CR3] American College of Physicians. 2022. Attacks on gender-affirming and transgender health care. https://www.acponline.org/advocacy/state-health-policy/attacks-on-gender-affirming-and-transgender-health-care. Accessed May 9, 2023.

[CR4] American Psychiatric Association. 2013. *Diagnostic and statistical manual of mental disorders*, 5th ed. Washington DC: American Psychiatric Association.

[CR5] Anjum, R.L., and S.D. Mumford. 2017. A philosophical argument against evidence-based policy. *Journal of Evaluation in Clinical Practice* 23(5): 1045–1050. 27282999 10.1111/jep.12578

[CR6] Ashley, F. 2019. Gatekeeping hormone replacement therapy for transgender patients is dehumanizing. *Journal of Medical Ethics* 45(7): 480–482. 30988174 10.1136/medethics-2018-105293

[CR7] Ashley, F. 2020. A critical commentary on “rapid-onset gender dysphoria.” *Sociological Review* 68(4): 779–799.

[CR8] Ashley, F. 2021. The clinical irrelevance of “desistance” research for transgender and gender creative youth. *Psychology of Sexual Orientation and Gender Diversity* 9(4): 387–397.

[CR9] ———. 2022. Adolescent medical transition is ethical: An analogy with reproductive health. *Kennedy Institute of Ethics Journal* 32(2): 127–171. 10.1353/ken.2022.0010 35815503

[CR10] Ashley, F., D.M. Tordoff, J. Olson-Kennedy, and A.J. Restar. 2023. Randomized-controlled trials are methodologically inappropriate in adolescent transgender healthcare. *International Journal of Transgender Health*, ePub ahead of print, June 24. 10.1080/26895269.2023.2218357. 10.1080/26895269.2023.2218357 PMC11268232 39055634

[CR11] Baker, J.T., B.R. Cusanno, and M. Dean. 2023. Dilemmas in patient-clinician communication about do-it-yourself hormone therapy: A qualitative study. *SSM – Qualitative Research in Health* 3: 100213.

[CR12] Baron, T., and G. Dierckxsens. 2022. Two dilemmas for medical ethics in the treatment of gender dysphoria in youth. *Journal of Medical Ethics* 48(9): 603–607. 34059519 10.1136/medethics-2021-107260

[CR13] Barratt, J. 2016. Doctors are failing to help people with gender dysphoria. *BMJ* 352: i694. 27030467 10.1136/bmj.i1694

[CR14] Beauchamp, T.L., and J.F. Childress. 2001. *The principles of biomedical ethics*, 5th ed. New York: Oxford University Press.

[CR15] Bell, K. 2021. Keira Bell: My story. *Persuasion*, April 7. https://www.persuasion.community/p/keira-bell-my-story. Accessed May 9, 2023.

[CR16] Bettcher, T.M. 2009. Trans identities and first-person authority. In *You’ve changed: Sex reassignment and personal identity*, edited by L. Shrage, 98–120. Oxford: Oxford University Press.

[CR17] Brik, T., L.J.J.J. Vrouenraets, M.C. de Vries, and S.E. Hannema. 2020. Trajectories of adolescents treated with gonadotropin-releasing hormone analogues for gender dysphoria. *Archives of Sexual Behavior* 49(7): 2611–2618. 32152785 10.1007/s10508-020-01660-8 PMC7497424

[CR18] Brown, C. 2021. Exploring trans people’s experiences of adoption and fostering in the United Kingdom: A qualitative study. *International Journal of Transgender Health *22(1–2): 89–100. 10.1080/26895269.2020.1867396 PMC8040684 33899048

[CR19] Bungener, S.L., A.L. C. de Vries, A. Popma, and T.D. Steensma. 2020. Sexual experiences of young transgender persons during and after gender-affirmative treatment. *Pediatrics* 146(6): e20191411. 33257402 10.1542/peds.2019-1411

[CR20] Carmichael, P., G. Butler, U. Masic, et al. 2021. Short-term outcomes of pubertal suppression in a selected cohort of 12 to 15 year old young people with persistent gender dysphoria in the UK. *PLoS ONE* 16(2): e0243894. 33529227 10.1371/journal.pone.0243894 PMC7853497

[CR21] Chen, D., J. Berona, Y.M. Chan, et al. 2023. Psychosocial functioning in transgender youth after 2 years of hormones. *New England Journal of Medicine* 388(3): 240–250. 36652355 10.1056/NEJMoa2206297 PMC10081536

[CR22] Cheng, P.J., A.W. Pastuszak, J.B. Myers, I.A. Goodwin, and J.M. Hotaling. 2019. Fertility concerns of the transgender patient. *Translational Andrology and Urology* 8(3): 209–218. 31380227 10.21037/tau.2019.05.09 PMC6626312

[CR23] Chew, D., J. Anderson, K. Williams, T. May, and K. Pang. 2018. Hormonal treatment in young people with gender dysphoria: A systematic review. *Pediatrics* 141(4): e20173742. 29514975 10.1542/peds.2017-3742

[CR24] Clark, B.A., and A. Virani. 2021. “This wasn’t a split-second decision”: An empirical ethical analysis of transgender youth capacity, rights, and authority to consent to hormone therapy. *Journal of Bioethical Inquiry* 18(1): 151–164. 33502682 10.1007/s11673-020-10086-9 PMC8043901

[CR25] Cohen-Kettenis, P.T., and S.H. van Goozen. 1997. Sex reassignment of adolescent transsexuals: A follow-up study. *Journal of the American Academy of Child and Adolescent Psychiatry* 36(2): 263–271. 10.1097/00004583-199702000-00017 9031580

[CR26] Cowley, C. 2005. The dangers of medical ethics. *Journal of Medical Ethics* 31(12): 739–742. 16319242 10.1136/jme.2005.011908 PMC1734063

[CR27] Cummings, C.L., and M.R. Mercurio. 2010. Ethics for the pediatrician: Autonomy, beneficence, and rights. *Pediatrics in Review* 31(6): 252–255. 20516238 10.1542/pir.31-6-252

[CR28] de Freitas, L.D., G. Léda-Rêgo, S. Bezerra-Filho, and Â. Miranda-Scippa. 2020. Psychiatric disorders in individuals diagnosed with gender dysphoria: A systematic review. *Psychiatry and Clinical Neurosciences* 74(2): 99–104. 10.1111/pcn.12947 31642568

[CR29] de Vries, A.L.C., J.K. McGuire, T.D. Steensma, E.C.F. Wagenaar, T.A.H. Doreleijers, and P. T. Cohen-Kettenis. 2014. Young adult psychological outcome after puberty suppression and gender reassignment. *Pediatrics* 134(4): 696–704. 25201798 10.1542/peds.2013-2958

[CR30] de Vries, A.L.C., T.D. Steensma, T.A.H. Doreleijers, and P.T. Cohen-Kettenis. 2011. Puberty suppression in adolescents with gender identity disorder: A prospective follow-up study. *Journal of Sexual Medicine* 8(8): 2276–2283. 20646177 10.1111/j.1743-6109.2010.01943.x

[CR31] Deaton, A., and N. Cartwright. 2018. Understanding and misunderstanding randomized controlled trials. *Social Science and Medicine* 210: 2–21. 29331519 10.1016/j.socscimed.2017.12.005 PMC6019115

[CR32] Dhejne, C., R. Van Vlerken, G. Heylens, and J. Arcelus. 2016. Mental health and gender dysphoria: A review of the literature. *International Review of Psychiatry* 28(1): 44–57. 26835611 10.3109/09540261.2015.1115753

[CR33] Drummond, K.D., S.J. Bradley, M. Peterson-Badali, and K.J. Zucker. 2008. A follow-up study of girls with gender identity disorder. *Developmental Psychology* 44(1): 34–45. 18194003 10.1037/0012-1649.44.1.34

[CR34] Fix-Cañas, L.F., L. Mathew, V. Reddy, M. Kavenagh, J. Pearce, and C. Soares. 2022. What impacts do legally established minimum ages for providing consent to sexual activity have on young people? *Our Voices*. https://www.our-voices.org.uk/news/2022/what-impacts-do-legally-established-minimum-ages-for-providing-consent-to-sexual-activity-have-on-young-people. Accessed 13 Dec 2023.

[CR35] Fleming, C. 2006. Young people and the Fraser guidelines: Confidentiality and consent. *The Obstetrician and Gynaecologist* 8(4): 235–239.

[CR36] Free, C. 2005. Advice about sexual health for young people. *BMJ* 330(7483): 107–108. 15649909 10.1136/bmj.330.7483.107 PMC544416

[CR37] Fricker, M., and K. Jenkins. 2017. Epistemic injustice, ignorance, and trans experiences. In *The Routledge Companion to Feminist Philosophy*, edited by A. Garry, S.J. Khader, and A. Stone, 268–278. London: Routledge.

[CR38] Frieden, T. R. 2017. Evidence for health decision making—Beyond randomized, controlled trials. *New England Journal of Medicine* 377(5): 465–475. 28767357 10.1056/NEJMra1614394

[CR39] Gillon, R. 1994. Medical ethics: Four principles plus attention to scope. *BMJ* 309(6948): 184–188. 8044100 10.1136/bmj.309.6948.184 PMC2540719

[CR40] Giordano, S., and S. Holm. 2020. Is puberty delaying treatment “experimental treatment”? *International Journal of Transgender Health* 21(2): 113–121. 33015663 10.1080/26895269.2020.1747768 PMC7430465

[CR41] Good Law Project. 2023. We are just people too. *Good Law Project*, February 21. https://goodlawproject.org/case/we-are-just-people-too/. Accessed May 9, 2023.

[CR42] Grannis, C., S.F. Leibowitz, S. Gahn, et al. 2021. Testosterone treatment, internalizing symptoms, and body image dissatisfaction in transgender boys. *Psychoneuroendocrinology* 132: e105358. 10.1016/j.psyneuen.2021.105358 34333318

[CR43] Green, A.E., J.P. DeChants, M.N. Price, and C.K. Davis. 2022. Association of gender-affirming hormone therapy with depression, thoughts of suicide, and attempted suicide among transgender and nonbinary youth. *Journal of Adolescent Health* 70(4): 643–649. 10.1016/j.jadohealth.2021.10.036 34920935

[CR44] Griffin, L., K. Clyde, R. Byng, and S. Bewley. 2021. Sex, gender and gender identity: a re-evaluation of the evidence. *BJPsych Bulletin* 45(5): 291–299. 32690121 10.1192/bjb.2020.73 PMC8596152

[CR45] Grossman, J., and F.J. Mackenzie. 2005. The randomized controlled trial: Gold standard, or merely standard? *Perspectives in Biology and Medicine* 48(4): 516–534. 16227664 10.1353/pbm.2005.0092

[CR46] Guyatt, G.H., A.D. Oxman, G.E. Vist, et al. 2008. GRADE: An emerging consensus on rating quality of evidence and strength of recommendations. *BMJ* 336(7650): 924–926. 18436948 10.1136/bmj.39489.470347.AD PMC2335261

[CR47] Hembree, W.C., P.T. Cohen-Kettenis, L. Gooren, et al. 2017. Endocrine treatment of gender-dysphoric/gender-incongruent persons: An endocrine society clinical practice guideline. *Journal of Clinical Endocrinology and Metabolism* 102(11): 3869–3903. 28945902 10.1210/jc.2017-01658

[CR48] Hines, S. 2020. Sex wars and (trans) gender panics: Identity and body politics in contemporary UK feminism. *Sociological Review* 68(4): 699–717.

[CR49] Home Office. 2004. *Children and families: Safer from sexual crime – The Sexual Offences Act 2003*. London: Home Office Communications Directorate.

[CR50] Hughto, J.M.W., S.L. Reisner, and J.E. Pachankis. 2015. Transgender stigma and health: A critical review of stigma determinants, mechanisms, and interventions. *Social Science and Medicine* 147: 222–231. 26599625 10.1016/j.socscimed.2015.11.010 PMC4689648

[CR51] Jackson, S.S., J. Brown, R.M. Pfeiffer, et al. 2023. Analysis of mortality among transgender and gender diverse adults in England. *JAMA Network Open* 6(1): e2253687. 36716027 10.1001/jamanetworkopen.2022.53687 PMC9887492

[CR52] Kahlenberg, C.A., B.U. Nwachukwu, A.S. McLawhorn, M.B. Cross, C.N. Cornell, and D.E. Padgett. 2018. Patient satisfaction after total knee replacement: A systematic review. *HSS Journal* 14(2): 192–201. 29983663 10.1007/s11420-018-9614-8 PMC6031540

[CR53] Kanbur, N. 2019. Close-in-age exemption laws: Focusing on the best interests of children and adolescents. *International Journal of Adolescent Medicine and Health* 33(2). 10.1515/ijamh-2018-0143. 10.1515/ijamh-2018-0143 31095508

[CR54] Katz-Wise, S.L., D. Ehrensaft, R. Vetters, M. Forcier, and S.B. Austin. 2018. Family functioning and mental health of transgender and gender-nonconforming youth in the trans teen and family narratives project. *Journal of Sex Research* 55(4–5): 582–590. 29336604 10.1080/00224499.2017.1415291 PMC7895334

[CR55] Klein, A., and S.A. Golub. 2016. Family rejection as a predictor of suicide attempts and substance misuse among transgender and gender nonconforming adults. *LGBT Health* 3(3): 193–199. 27046450 10.1089/lgbt.2015.0111

[CR56] Klink, D., M. Caris, A. Heijboer, M. van Trotsenburg, and J. Rotteveel. 2015. Bone mass in young adulthood following gonadotropin-releasing hormone analog treatment and cross-sex hormone treatment in adolescents with gender dysphoria. *Journal of Clinical Endocrinology and Metabolism* 100(2): e270–e275. 25427144 10.1210/jc.2014-2439

[CR57] Kotamarti, V.S., N. Greige, A.J. Heiman, A. Patel, and J.A. Ricci. 2021. Risk for venous thromboembolism in transgender patients undergoing cross-sex hormone treatment: A systematic review. *Journal of Sexual Medicine* 18(7): 1280–1291. 34140253 10.1016/j.jsxm.2021.04.006

[CR58] Kraschel, K.L., A. Chen, J.L. Turban, and I.G. Cohen. 2022. Legislation restricting gender-affirming care for transgender youth: Politics eclipse healthcare. *Cell Reports Medicine* 3(8): e100719. 10.1016/j.xcrm.2022.100719 PMC9418844 35977463

[CR59] Kuper, L.E., S. Stewart, S. Preston, M. Lau, and X. Lopez. 2020. Body dissatisfaction and mental health outcomes of youth on gender affirming hormone therapy. *Pediatrics* 145(4): e20193006. 32220906 10.1542/peds.2019-3006

[CR60] Larcher, V., and A. Hutchinson. 2010. How should paediatricians assess Gillick competence? *Archives of Disease in Childhood* 95(4): 307–311. 19948515 10.1136/adc.2008.148676

[CR61] Latham, A. 2022. Puberty blockers for children: Can they consent? *The New Bioethics* 28(3): 268–291. 35758886 10.1080/20502877.2022.2088048

[CR62] Littman, L., 2018. Parent reports of adolescents and young adults perceived to show signs of a rapid onset of gender dysphoria. *PLoS One* 13(8): e0202330. 30114286 10.1371/journal.pone.0202330 PMC6095578

[CR63] ———. 2019. Correction: Parent reports of adolescents and young adults perceived to show signs of a rapid onset of gender dysphoria. *PLoS One* 14(3): e0214157. 10.1371/journal.pone.0214157 PMC6424391 30889220

[CR64] MacKinnon, K.R., H. Kia, T. Salway, et al. 2022. Health care experiences of patients discontinuing or reversing prior gender-affirming treatments. *JAMA Network Open* 5(7): e2224717. 35877120 10.1001/jamanetworkopen.2022.24717 PMC9315415

[CR65] Madsen, M.C., D. van Dijk, C.M. Wiepjes, E.B. Conemans, A. Thijs, and M. den Heijer. 2021. Erythrocytosis in a large cohort of trans men using testosterone: A long-term follow-up study on prevalence, determinants, and exposure years. *Journal of Clinical Endocrinology and Metabolism* 106(6): 1710–1717. 33599731 10.1210/clinem/dgab089 PMC8118580

[CR66] Mahfouda, S., J.K. Moore, A. Siafarikas, F.D. Zepf, and A. Lin. 2017. Puberty suppression in transgender children and adolescents. *Lancet Diabetes and Endocrinology* 5(10): 816–826. 28546095 10.1016/S2213-8587(17)30099-2

[CR67] Malatino, H. 2020. *Trans Care*. Minneapolis: University of Minnesota Press.

[CR68] Mason, A., E. Crowe, B. Haragan, S. Smith, and A. Kyriakou. 2023. Gender dysphoria in young people: A model of chronic stress. *Hormone Research in Paediatrics* 96(1): 54–65. 34673639 10.1159/000520361

[CR69] Maung, H.H. 2019. Is infertility a disease and does it matter? *Bioethics* 33(1): 43–53. 30106176 10.1111/bioe.12495 PMC6492453

[CR70] McDougall, R., L. Notini, C. Delany, M. Telfer, and K.C. Pang. 2021. Should clinicians make chest surgery available to transgender male adolescents? *Bioethics* 35(7): 696–703. 34196960 10.1111/bioe.12912

[CR71] McLean, C. 2021. The growth of the anti-transgender movement in the United Kingdom. The silent radicalization of the British electorate. *International Journal of Sociology* 51(6): 473–482.

[CR72] Mitchell E.A., R. Scragg, A.W. Stewart, et al. 1991. Results from the first year of the New Zealand cot death study. *New Zealand Medical Journal* 104(906): 71–76. 2020450

[CR73] Moore, A.M. 2018. Health related quality of life of transgender adolescents undergoing hormonal transition or elective pubertal delay. Doctor of Medicine thesis, University of Texas Southwestern Medical Center.

[CR74] Murphy, T.F. 2019. Adolescents and body modification for gender identity expression. *Medical Law Review* 27(4): 623–639. 31004152 10.1093/medlaw/fwz006 PMC6926440

[CR75] National Institute for Health and Care Excellence. 2022a. Evidence review: Gender-affirming hormones for children and adolescents with gender dysphoria. London: National Institute for Health and Care Excellence. https://perma.cc/9KLH-PJFG. Accessed May 9, 2023.

[CR76] ———. 2022b. Evidence review: Gonadotrophin releasing hormone analogues for children and adolescents with gender dysphoria. London: National Institute for Health and Care Excellence. https://perma.cc/TBG4-AF9Z. Accessed May 9, 2023.

[CR77] NHS England. 2022. Interim service specification for specialist gender dysphoria services for children and young people. London: NHS England. https://www.engage.england.nhs.uk/specialised-commissioning/gender-dysphoria-services/user_uploads/b1937-ii-interim-service-specification-for-specialist-gender-dysphoria-services-for-children-and-young-people-22.pdf. Accessed May 9, 2023.

[CR78] ———. 2023. Interim specialist service for children and young people with gender incongruence. London: NHS England. https://www.england.nhs.uk/wp-content/uploads/2023/06/Interim-service-specification-for-Specialist-Gender-Incongruence-Services-for-Children-and-Young-People.pdf. Accessed August 10, 2023.

[CR79] Olson, K.R., L. Durwood, R. Horton, N. M. Gallagher, and A. Devor. 2022. Gender identity 5 years after social transition. *Pediatrics *150(2): e2021056082. 10.1542/peds.2021-056082 PMC9936352 35505568

[CR80] Paul, L.A. 2014. *Transformative experience*. Oxford: Oxford University Press.

[CR81] Pearce, N., and J.P. Vandenbroucke. 2021. Arguments about face masks and Covid-19 reflect broader methodologic debates within medical science. *European Journal of Epidemiology* 36(2): 143–147. 33725291 10.1007/s10654-021-00735-7 PMC7961168

[CR82] Ratcliff, C.G., L. Cohen, C.A. Pettaway, and P.A. Parker. 2013. Treatment regret and quality of life following radical prostatectomy. *Supportive Care in Cancer* 21(12): 3337–3343. 23907238 10.1007/s00520-013-1906-4 PMC3823814

[CR83] Restar, A.J. 2020. Methodological critique of Littman’s (2018) Parental-respondents accounts of “rapid-onset gender dysphoria.” *Archives of Sexual Behavior* 49(1): 61–66. 31011991 10.1007/s10508-019-1453-2 PMC7012957

[CR84] Rew, L., C.C. Young, M. Monge, and R. Bogucka. 2021. Review: Puberty blockers for transgender and gender diverse youth—A critical review of the literature. *Child and Adolescent Mental Health* 26(1): 3–14. 33320999 10.1111/camh.12437

[CR85] Richards, C., J. Maxwell, and N. McCune. 2019. Use of puberty blockers for gender dysphoria: A momentous step in the dark. *Archives of Disease in Childhood* 104(6): 611–612. 30655265 10.1136/archdischild-2018-315881

[CR86] Rider, G.N., B.J. McMorris, A.L. Gower, E. Coleman, and M.E. Eisenberg. 2018. Health and care utilization of transgender and gender nonconforming youth: A population-based study. *Pediatrics* 141(3): e20171683. 29437861 10.1542/peds.2017-1683 PMC5847087

[CR87] Russo, F., and J. Williamson. 2007. Interpreting causality in the health sciences, *International Studies in the Philosophy of Science* 21(2): 157–170.

[CR88] Safer, J.D., E. Coleman, J.F.R. Garofalo, W.C. Hembree, A. Radix, and J. Sevelius. 2016. Barriers to health care for transgender individuals. *Current Opinion in Endocrinology, Diabetes, and Obesity* 23(2): 168–171. 26910276 10.1097/MED.0000000000000227 PMC4802845

[CR89] Schagen, S.E.E., F.M. Wouters, P.T. Cohen-Kettenis, L.J. Gooren, and S.E. Hannema. 2020. Bone development in transgender adolescents treated with GnRH analogues and subsequent gender-affirming hormones. *Journal of Clinical Endocrinology and Metabolism* 105(12): e4252–e4263. 32909025 10.1210/clinem/dgaa604 PMC7524308

[CR90] Schulz, S.L. 2018. The informed consent model of transgender care: An alternative to the diagnosis of gender dysphoria. *Journal of Humanistic Psychology* 58(1): 72–92.

[CR91] Singal, J. 2018. When children say they’re trans. *The Atlantic*, July/August. https://www.theatlantic.com/magazine/archive/2018/07/when-a-child-says-shes-trans/561749/. Accessed May 9, 2023.

[CR92] Sorbara, J.C., L.N. Chiniara, S. Thompson, and M.R. Palmert. 2020. Mental health and timing of gender-affirming care. *Pediatrics* 146(4): e20193600. 32958610 10.1542/peds.2019-3600

[CR93] Staples, J.M., E.R. Bird, T.N. Masters, and W.H. George. 2018. Considerations for culturally sensitive research with transgender adults: A qualitative analysis. *Journal of Sex Research* 55(8): 1065–1076. 10.1080/00224499.2017.1292419 PMC7064308 28276930

[CR94] Swedish National Board of Health and Welfare. 2022. *Care of children and adolescents with gender dysphoria*. Stockholm: Swedish National Board of Health and Welfare. https://www.socialstyrelsen.se/globalassets/sharepoint-dokument/artikelkatalog/kunskapsstod/2022-3-7799.pdf. Accessed May 9, 2023.

[CR95] Tan, K.K.H., J.L. Byrne, G.J. Treharne, and J.F. Veale. 2023. Unmet need for gender-affirming care as a social determinant of mental health inequities for transgender youth in Aotearoa/New Zealand. *Journal of Public Health* 45(2): e225–e233. 36468999 10.1093/pubmed/fdac131 PMC10273389

[CR96] Temple Newhook, J., J. Pyne, K. Winters, et al. 2018. A critical commentary on follow-up studies and “desistance” theories about transgender and gender-nonconforming children. *International Journal of Transgenderism* 19(2): 212–224.

[CR97] Tordoff, D.M., J.W. Wanta, A. Collin, C. Stepney, D.J. Inwards-Breland, and K. Ahrens. 2022. Mental health outcomes in transgender and nonbinary youths receiving gender-affirming care. *JAMA Network Open* 5(2): e220978. 35212746 10.1001/jamanetworkopen.2022.0978 PMC8881768

[CR98] Trevor Project. 2020. *National survey on LGBTQ youth mental health 2020*. https://www.thetrevorproject.org/survey-2020/. Accessed August 17, 2023.

[CR99] Turban, J.L., D. King, J.M. Carswell, and A.S. Keuroghlian. 2020. Pubertal suppression for transgender youth and risk of suicidal ideation. *Pediatrics* 145(2): e20191725. 31974216 10.1542/peds.2019-1725 PMC7073269

[CR100] Turban, D.L., S.S. Loo, A. N. Almazan, and A.S. Keuroghlian. 2021. Factors leading to “detransition” among transgender and gender diverse people in the United States: A mixed-methods analysis. *LGBT Health* 8(4): 273–280. 33794108 10.1089/lgbt.2020.0437 PMC8213007

[CR101] Turner, L. 2003. Bioethics in a multicultural world: Medicine and morality in pluralistic settings. *Health Care Analysis* 11(2): 99–117. 14567474 10.1023/A:1025620211852

[CR102] van der Loos, M.A.T.C., S.E. Hannema, D.T. Klink, M. den Heijer, and C.M. Wjepjes. 2022. Continuation of gender-affirming hormones in transgender people starting puberty suppression in adolescence: A cohort study in the Netherlands. *Lancet Child and Adolescent* 6(12): 869–875. 10.1016/S2352-4642(22)00254-1 36273487

[CR103] van der Miesen, A., T.D. Steensma, A. de Vries, H. Bos, and A. Popma. 2020. Psychological functioning in transgender adolescents before and after gender-affirmative care compared with cisgender general population peers. *Journal of Adolescent Health* 66(6): 699–704. 10.1016/j.jadohealth.2019.12.018 32273193

[CR104] Varkey, B. 2021. Principles of clinical ethics and their application to practice. *Medical Principles and Practice* 30(1): 17–28. 32498071 10.1159/000509119 PMC7923912

[CR105] Vlot, M.C., D.T. Klink, M. den Heijer, M.A. Blankenstein, J. Rotteveel, and A.C. Heijboer. 2017. Effect of pubertal suppression and cross-sex hormone therapy on bone turnover markers and bone mineral apparent density (BMAD) in transgender adolescents. *Bone* 95: 11–19. 27845262 10.1016/j.bone.2016.11.008

[CR106] Wallien, M.S.C., and P.T. Cohen-Kettenis. 2008. Psychosexual outcome of gender-dysphoric children. *Journal of the American Academy of Child and Adolescent Psychiatry* 47(12): 1413–1423. 18981931 10.1097/CHI.0b013e31818956b9

[CR107] Watson, L. 2016. The woman question. *Transgender Studies Quarterly* 3(1–2): 246–253.

[CR108] Weiss, J.T. 2001. The gender caste system: Identity, privacy and heteronormativity. *Law and Sexuality* 10: 123–186.

[CR109] Williams, J. 2020. Our approach to children and gender is a moral outrage. *The Telegraph*, January 6. https://www.telegraph.co.uk/news/2020/01/06/approach-gender-self-id-moral-outrage/. Accessed May 9, 2023.

[CR110] Woodward, J. 2003. *Making things happen: A theory of causal explanation*. Oxford: Oxford University Press.

[CR111] World Health Organization. 2019. *International classification of diseases*, 11th revision. Geneva: World Health Organization.

[CR112] World Professional Association for Transgender Health. 2022a. Standards of care for the health of transgender and gender diverse people, version 8. *International Journal of Transgender Health* 23: S1–S259. 36238954 10.1080/26895269.2022.2100644 PMC9553112

[CR113] ———. 2022b. Statement regarding the Interim Service Specification for the Specialist Service for Children and Young People with Gender Dysphoria (Phase 1 Providers) by NHS England, 25 November. https://www.wpath.org/media/cms/Documents/Public%20Policies/2022/25.11.22%20AUSPATH%20Statement%20reworked%20for%20WPATH%20Final%20ASIAPATH.EPATH.PATHA.USPATH.pdf?_t=1669428978. Accessed May 9, 2023.

[CR114] Yeh, R.W., L.R. Valsdottir, M.W. Yeh, et al. 2018. Parachute use to prevent death and major trauma when jumping from aircraft: Randomized controlled trial. *BMJ* 13: k5094. 10.1136/bmj.k5094 PMC6298200 30545967

[CR115] Zwickl, S., A.F.Q. Wong, E. Dowers, et al. 2021. Factors associated with suicide attempts among Australian transgender adults. *BMC Psychiatry* 21(1): 81. 33557793 10.1186/s12888-021-03084-7 PMC7869522

[CR116] Zucker, K.J., and S.J. Bradley. 1995. *Gender identity disorder and psychosexual problems in children and adolescents*. New York: Guilford Press. 10.1177/070674379003500603 2207982

